# An Interesting Case of Inherited Syphilis

**Published:** 1916-02

**Authors:** A. H. Lindorme

**Affiliations:** Major Medical Corps National Guard, Georgia


### AN INTERESTING CASE OF INHERITED SYPHILIS.

By A. H. Lindorme, M. D., Major Medical Corps National Guard, Georgia.

On Aug. 14th, 1914, I was called to attend a little girl, ten years old,—who had been—presumably struck by lightning. When I reached her, she was screaming with fright, and complained of pain, but could give no definite answer as to where the pain was.

This history, I gathered from the other children playing with her: they were playing in the swing under the trees when a storm came up. An exceedingly loud clap of thunder startled them and they all started to run to the house. As my patient ran, her left leg suddenly gave way and she fell to the ground unable to rise. She was carried into the house by the other children, and I was called,—their family physician being out of reach just then.

On Sept. 6th, the case was turned over to me. The father of the child, who is a brother officer in my Regiment, finally admitted to the following history:

In the summer of 1893 he was infected· The infection was diagnosed as syphilis, though having but few of the symptoms. He says there was no eruption, no sore throat; but there was a single ulcer which was considered typical. He says that he was under treatment for three or four months. For some years after he took a "blood medicine" every spring which contained iodid of potash.

In the year 1901, his lips became very sore and swollen. The symptoms subsided under anti-syphilitic treatment.

He was married in 1902. The first child, a girl, and the patient in this article, was born about three years after. She was a vigorous and healthy baby. When she was about four years old, she had a severe illness, which was said to have been Typhoid Fever, but after several weeks' illness she recovered entirely.

Three and one-half years later, a boy was born. Three years subsequent, another boy was born, both seemingly healthy children at birth.

When the child was about two years old, the mother had an abortion of about three months. From what I can learn, I do not believe that this happened as a consequence of syphilis. The wife and mother seems to have escaped all infection.

The first born, the little girl, when ten years old, as stated in the beginning of this paper, was supposed to have been struck or shocked by lightening. On my questioning the mother, I learned that the child's condition from the time I first saw her up to now, was as follows: From once to twice each day she would have one of her "spells", the nature of which will be described fully later on.

She ran a temperature of from 101 to 102 1-2, with loss of appetite, persistent constipation, foul breath, coated tongue and "rheumatic pains" in her shoulders, arms, and sometimes down her legs, and she also suffered with severe headache. Her lips were covered with Herpes.

To relieve these "rheumatic pains" she was given five grain doses of aspirin, so the mother told me, but with very little amelioration of symptoms.

On making further examinations, I learned that she either could not, or would not try to stand. The left leg remained flexed on account of the contraction of the flexor muscles of the thigh.

In the left popliteal space was a deep seated abscess, with a number of smaller ulcers and abscesses on the leg and ankle.

She did not use her left arm, we noticed that she picked up articles with her right hand.

I could not make an examination of the tendon reflexes on account of her hysterical condition and fear on the approach of anyone who endeavored to move her limbs.

The "spells" were a condition which would be precipitated at any time if the patient was crossed in any of her wishes or desires.

Then she would jerk all over, throw herself from one side of the bed to the other, moaning as if in pain, and calling for her mother to save her from some impending danger.

A few times, these attacks were severe and she was unconscious for some minutes. After the attacks would wear off, she would lie in a dazed condition, and was very much exhausted. Her mind suffered also. She was unreasonable in the extreme, and only the gentlest and most persuasive means could be used. She would never do anything except that she thought it was by her own wish.

On Sept. 10th, the laboratory reported to me its findings of a positive Wasserman reaction. On the strength of this report, anti-syphlitic treatment was vigorously pushed.

Five minims of a saturated solution of iodid of potash was given three times a day. After a few days it was increased at the rate of 1 minim at each dose until 40 minims were given at a time.

At this high dosage, we began to get some headache and a watery discharge from the nose. In other words, the effect of too much iodid. The dose was then reduced to fifteen minims and then gradually raised to 30 minims.

Leaving off the iodid of potash, entirely and giving 1-100 of a grain of bichloride of mercury, I gradually ran the dose up to 1-60, but this began to give us trouble. After about two weeks, I noticed a redness and tenderness of the gums. Then she was put back on the iodids.

This heroic treatment had its beneficial effect. She made a steady improvement. Her "spells" came less frequently, were a great deal milder, and her whole disposition improved.

The abscess and sores healed. Her appetite improved, and she slept better and began to take interest in everything.

About the first of October a faradic battery was used. Putting her feet on a copper plate and a sponge electro was passed up and down her spine. This treatment was given in the evening for about twenty minutes duration.

The first part of October, I was very much surprised one night when calling at the home of my little patient, to be met at the door by her. She was rather unsteady on her feet, and not using her left leg well, dragging it with the toe inclined to remain on the floor. A steady improvement in her walking was noticeable, however, and the young lady herself made every effort to again walk naturally.

School had started for the fall term, and she was anxious to go, but I feared that the mental effort would be too much for her. Persistent begging finally won us over, and she started to school. She attended the afternoon session, which was of only four hours duration. At first, she came home very tired and irritable, but this rapidly passed and before the term was over, she repeatedly led her class.

The most interesting features in this case are, to my mind: (1) The seemingly mild infection of the father and the short time of his treatment.

(2) The length of time, twelve months, to the birth of the child, the patient in this case, and again, ten years later, the onset of the symptoms of syphilis in the child.

(3) Was it severe fright, or was it the electric shock which precipitated this attack?

A. H. LINDORME,

